# p38 MAPK Signaling in Postnatal Tendon Growth and Remodeling

**DOI:** 10.1371/journal.pone.0120044

**Published:** 2015-03-13

**Authors:** Andrew J. Schwartz, Dylan C. Sarver, Kristoffer B. Sugg, Justin T. Dzierzawski, Jonathan P. Gumucio, Christopher L. Mendias

**Affiliations:** 1 Department of Orthopaedic Surgery, University of Michigan Medical School, Ann Arbor, Michigan, United States of America; 2 Department of Molecular & Integrative Physiology, University of Michigan Medical School, Ann Arbor, Michigan, United States of America; 3 Department of Surgery, Section of Plastic Surgery, University of Michigan Medical School, Ann Arbor, Michigan, United States of America; University of Birmingham, UNITED KINGDOM

## Abstract

Tendon is a dynamic tissue whose structure and function is influenced by mechanical loading, but little is known about the fundamental mechanisms that regulate tendon growth and remodeling *in vivo*. Data from cultured tendon fibroblasts indicated that the p38 MAPK pathway plays an important role in tendon fibroblast proliferation and collagen synthesis *in vitro*. To gain greater insight into the mechanisms of tendon growth, and explore the role of p38 MAPK signaling in this process, we tested the hypotheses that inducing plantaris tendon growth through the ablation of the synergist Achilles tendon would result in rapid expansion of a neotendon matrix surrounding the original tendon, and that treatment with the p38 MAPK inhibitor SB203580 would prevent this growth. Rats were treated with vehicle or SB203580, and subjected to synergist ablation by bilateral tenectomy of the Achilles tendon. Changes in histological and biochemical properties of plantaris tendons were analyzed 3, 7, or 28 days after overload, and comparisons were made to non-overloaded animals. By 28 days after overload, tendon mass had increased by 30% compared to non-overloaded samples, and cross-sectional area (CSA) increased by around 50%, with most of the change occurring in the neotendon. The expansion in CSA initially occurred through the synthesis of a hyaluronic acid rich matrix that was progressively replaced with mature collagen. Pericytes were present in areas of active tendon growth, but never in the original tendon ECM. Inhibition of p38 MAPK resulted in a profound decrease in IL6 expression, and had a modest effect on the expression of other ECM and cell proliferation genes, but had a negligible impact on overall tendon growth. The combined results from this study provided novel insights into tendon mechanobiology, and suggest that p38 MAPK signaling does not appear to be necessary for tendon growth *in vivo*.

## Introduction

Tendon plays a vital role in the musculoskeletal system by transmitting forces between skeletal muscle and bone. Tendon is composed predominately of a dense extracellular matrix (ECM) of type I collagen, but also type III collagen, elastin, and various proteoglycans and glycoproteins [1]. Injuries and chronic degenerative conditions of tendon are among the more common musculoskeletal morbidities, but current treatment options for tendinopathies are quite limited [2]. This is particularly true for tendinosis, which is a chronic, painful overuse condition thought to be caused by a failure of tendon fibroblasts to properly regenerate the ECM after mechanical load-induced injury [1, 2]. A major limitation in our ability to treat tendinopathies is our lack of knowledge about the basic biological mechanisms of tendon growth and remodeling, and gaining further insight into these processes is likely to improve regenerative medicine strategies for the treatment of tendon injuries and diseases.

Much of our understanding of the in vivo cellular and molecular mechanisms of tendon growth comes from the developmental biology literature. Transforming growth factor-beta (TGF-b) directs the expression of several genes that regulate tendon fibroblast proliferation and ECM synthesis, and mice that lack the type II TGF-b receptor in limb bud mesenchyme fail to form tendons [3]. One of the more important downstream targets of TGF-b in tendon fibroblasts is the basic helix-loop-helix (bHLH) transcription factor scleraxis, which directs the expression of several genes involved in tendon growth and maturation during the later stages of embryogenesis [3–5]. TGF-b, and the closely related signaling molecule myostatin, can also induce scleraxis expression in adult tendon fibroblasts and promote cell proliferation and ECM synthesis [6, 7]. Signaling from TGF-b can activate the canonical Smad2/3 transcription factor pathway, as well as the non-canonical p38 MAPK intracellular signaling pathway [8]. Using in vitro studies, we previously reported that blocking the p38 MAPK pathway had a more profound inhibitory effect on adult tendon fibroblast proliferation and type I collagen synthesis than inhibition of the Smad2/3 pathway [7], suggesting that p38 MAPK may play an important role in TGF-b superfamily mediated growth and remodeling of tendon. Mechanical loading also appears to be an important stimulus for the induction of scleraxis expression in adult tendon [6, 9, 10], and this process is likely due at least in part to TGF-b signaling [11]. However, to our knowledge, the role of p38 MAPK signaling in the growth and remodeling of adult tendon to a mechanical stimulus has not previously been evaluated in vivo.

The hindlimb synergist ablation model, in which a tenotomy of the Achilles tendon is performed leaving the plantaris muscle as the sole ankle plantarflexor, has proven to be an invaluable technique in the study of skeletal muscle growth [12–18]. Recently we demonstrated that this model, when used in mice, also leads to rapid and robust tendon growth [9]. While murine models offer several advantages to study basic tendon physiology, such as highly homogenous strains that reduce the effect of genetic variation on the observed phenotypes that arise following experimental interventions, they are limited by the relatively small size of tendons and the subsequent small substrates available for biochemical and histological analyses. Outbred strains of rats are 10 to 30 times larger than mice, have more diverse genetic make-up, and have greater sophistication in the anatomical ultrastructure and mechanical properties of tendons, which enhances the ability of rat models to be used for translational studies [19, 20]. With this in mind, we sought to determine if the synergist ablation model of plantaris tendon hypertrophy we previously reported in mice [9] was also applicable to the study of tendon growth in rats. As a second objective, since in vitro studies suggest that p38 MAPK is likely important in the biology of tendon fibroblasts, we investigated if blocking p38 MAPK signaling through the use of the small molecule SB203580 would alter tendon growth. We tested the hypothesis that the induction of tendon growth through the ablation of the synergist gastrocnemius and soleus muscles would result in a rapid growth and expansion of a neotendon matrix surrounding the original tendon, and that this neotendon matrix would be slowly replaced by a mature, type I collagen rich ECM. Further, we tested the hypothesis that treatment with SB203580 would prevent the normal growth and adaptation of tendon subjected to mechanical overload.

## Methods

### Ethics Statement

The University of Michigan Committee on the Use and Care of Animals approved this study (Protocol # PRO00003566). The humane care of animals followed the guidelines set forth in the US Public Health Service Policy on Humane Care and Use of Laboratory Animals. Surgeries were performed under appropriate anesthesia, and all efforts were made to minimize distress and pain.

### Surgical Procedure

Six-month-old male Sprague-Dawley rats were purchased from Charles River (Wilmington, MA) and housed under specific pathogen free conditions. For the main study, rats were randomized to 3, 7, or 28 day groups (N = 48 rats total, 16 in each group). Within each group, rats were randomized to receive treatment with a vehicle (N = 8) or SB203580 (N = 8) (Alfa Aesar, Ward Hill, MA), a specific inhibitor of p38 MAPK [21]. SB203580 was dissolved in dimethyl sulfoxide (DMSO), diluted in phosphate buffered saline (PBS), and administered via intraperitoneal injection at a dose of 3mg/kg one day prior to surgical intervention and 1mg/kg daily for seven days following surgical intervention. This dose was selected based on the work of Li [22], and pilot studies described below verified the ability of SB203580 to prevent p38 MAPK phosphorylation in vivo. Vehicle treated rats were administered a volume equivalent dose of DMSO in PBS. Rats were treated up to 7 days after overload based on previous results from mice [9] that demonstrated much of the change in total tendon CSA following synergist ablation occurs by this time point.

Rats were anesthetized with 2% isoflurane, and the skin overlying the surgical site was shaved and scrubbed with 4% chlorhexidine. A midline incision was created in the skin and the paratenon was split to achieve visualization of the Achilles tendon. A full-thickness tenectomy was performed in the mid-substance of the tendon as previously described [9, 12], and the entire midsubstance of the Achilles was removed to prevent spontaneous healing. The plantaris tendon was left intact. The paratenon was loosely re-approximated, a splash block of 0.5% bupivacaine was applied, and the skin was closed using 4–0 Vicryl (Ethicon, Somerville, NJ) and GLUture (Abbott, Abbott Park, IL). Ampicillin (20mg/kg), buprenorphine (0.03 mg/kg), and carprofen (5 mg/kg) were administered for analgesia and to prevent infection during post-operative recovery. Ad libitum weight bearing and cage activity were allowed, and rats were closely monitored for signs of pain or distress.

At harvest, rats were anesthetized with sodium pentobarbital (50mg/kg) and plantaris tendons were harvested and processed for immunoblotting, histology, hydroxyproline assay, or gene expression analysis. To provide reference data, plantaris tendons were also collected from age-matched control rats that were not subjected to synergist ablation. After tendons were removed, animals were humanely euthanized by anesthetic overdose and induction of bilateral pneumothorax.

### Immunoblots and SB203580 Dose Validation

**Fig 1:**
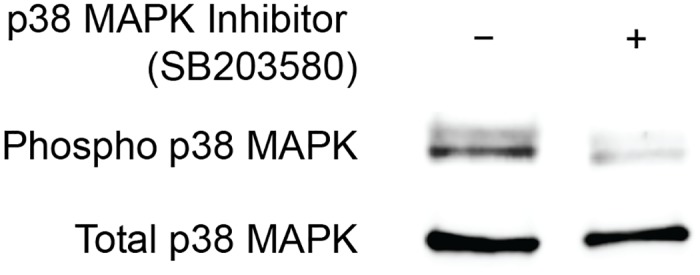
p38 MAPK blot. Representative immunoblot of overloaded plantaris tendons demonstrating the ability of SB203580 to prevent the phosphorylation of p38 MAPK. Total p38 MAPK protein is shown as a loading control.

While SB203580 has previously been used to block p38 MAPK activation in other tissues in vivo, to verify that SB203580 is effectively able to block p38 MAPK in tendon, rats received a single 3mg/kg dose of SB203580 or vehicle as described above. Plantaris overload surgeries were performed, and tissue was collected for analysis 6 hours later. Overloaded plantaris tendons were snap frozen in liquid nitrogen and made into a fine powder with a mortar and pestle. The tendon was further homogenized in 400μL of T-PER (Thermo Scientific, Waltham, MA) with a 1:100 protease and phosphatase inhibitor cocktail (Thermo Scientific). Protein concentration was determined using a Bradford protein assay (Thermo Scientific), and 7.5 μg of protein was diluted in Laemmli sample buffer with 1:20 beta-mercaptoethanol, placed in boiling water for 2 minutes, and then loaded into AnyKD mini-gels (BioRad, Berkeley, CA). Proteins were separated, then transferred from gels onto 0.45 μm nitrocellulose membranes (BioRad) using electrophoresis, blocked with 1% goat serum, and incubated with primary antibodies against either p38 MAPK or phospho-p38 MAPK (Cell Signaling Technology, Beverly, MA). After primary antibody incubation, membranes were rinsed and incubated with HRPO-conjugated goat anti-rabbit secondary antibodies (Abcam, Cambridge, MA). Proteins were detected using enhanced chemiluminescent reagents (BioRad) and visualized using a digital chemiluminescent documentation system (BioRad). Catalog numbers and dilutions of antibodies are listed in Table 1. Representative results are shown in Fig. 1.

**Table 1:** Immunoblot and immunohistochemistry detection reagents used in this study.

*Antibody*	*Manufacturer (Catalog #)*	*Dilution*
Goat anti rabbit HRPO	Abcam (#97057)	1:3000
Rabbit anti CD146	Abcam (#75769)	1:100
Rabbit anti Ki67	Abcam (#16667)	1:100
Rabbit anti F4/80, biotinylated	Abcam (#15694)	1:100
Rabbit anti p38 MAPK	Cell Signaling Technology (#8690)	1:2000
Rabbit anti phospho-p38 MAPK	Cell Signaling Technology (#4631)	1:2000
Hyaluronic acid binding protein, biotinylated	Millipore (#385911)	1:300
Goat anti rabbit, AF555	Life Technologies (A-21429)	1:200
Streptavidin, AF555	Life Technologies (S32355)	1:300
Rabbit anti procollagen type 1	Santa Cruz Biotechnology (#30136)	1:200

### Histology

The distal half of the right plantaris tendon was isolated, placed in TFM (Triangle Biosciences, Durham, NC), snap frozen in liquid nitrogen cooled isopentane, and stored at -80°C until use. Tendons were sectioned at a thickness of 10 μm in a cryostat. Sections were stained with hematoxylin and eosin (H&E) to determine CSA and cell density, or with picrosirius red (Polysciences Inc., Warrington, PA) to label collagen. High-resolution digital images were captured using an Olympus BX-51 microscope and camera. All quantification was performed using ImageJ software (NIH, Bethesda, MD) as described [7, 9].

For immunohistochemistry, slides were fixed in 4% paraformaldehyde, permeabilized in 0.4% Triton X-100, and blocked with 5% goat serum. Slides were incubated with primary anti- bodies against rabbit anti-Ki67 (Abcam), rabbit anti-CD146 (Abcam), biotinylated rabbit anti-F4/80 (Abcam), rabbit anti-procollagen type I (Santa Cruz Biotechnology, Dallas, TX), or biotinylated hyaluronic acid binding protein (HABP, Millipore, Billerica, MA). Secondary antibodies or streptavidin conjugated to AlexaFluor 555 (Life Technologies, Carlsbad, CA) were used to detect primary antibodies or HABP. Catalog numbers and dilutions of antibodies are provided in Table 1. DAPI (Sigma Aldrich, St. Louis, MO) was used to identify nuclei. Slides were mounted in Prolong Gold fluorescent mounting media (Life Technologies) and imaged using a Zeiss Axiovert 200M outfitted with the ApoTome system (Carl Zeiss, Thornwood, NY).

### Hydroxyproline Assay

The hydroxyproline content of the proximal half of the right plantaris tendon of rats was measured as described [23]. Briefly, tissue was dried at 110°C for one hour and then digested in 500μL 6N HCl at 130°C for six hours, followed by neutralization in an equal volume of 6N NaOH. The hydroxyproline in each sample was oxidized with chloramine-T and treated with dimethylaminobenzaldehyde (DMAB), which results in a colorimetric product that is proportional to the hydroxyproline quantity [24]. Absorbance values at 560nm were measured in a plate reader (Molecular Devices, Sunnyvale, CA).

### Gene Expression

Gene expression was performed as previously described [25, 26]. The left plantaris tendon was homogenized in QIAzol (Qiagen, Valencia, CA). RNA was isolated using a miRNeasy Kit (Qiagen), treated with DNase I (Qiagen), and reverse transcribed into cDNA using oligo-dT_15_ and random hexamer primers with a RT2 kit (Qiagen). cDNA was amplified in a CFX96 real-time thermal cycler (BioRad) using QuantiTect SYBR Green reagents (Qiagen). Target gene expression was normalized to the stable housekeeping gene beta 2-microglobulin (B2M), and further normalized to the relative expression values from plantaris tendons that were not subjected to synergist ablation using the 2^-ddCt^ technique [27]. Using this approach, any expression value greater than 1 indicates an upregulation compared to non-overloaded controls, and any value below 1 indicates a downregulation compared to controls. The list of genes and RefSeq ID numbers is provided in S1 Table.

### Statistical Analyses

Results are presented as mean±SD. Prism 6.0 software (GraphPad Software, La Jolla, CA) was used to conduct analyses. A two-way ANOVA (alpha = 0.05) followed by Newman-Keuls post-hoc sorting was performed to evaluate the interaction between time after overload and SB203580 treatment.

## Results

**Fig 2:**
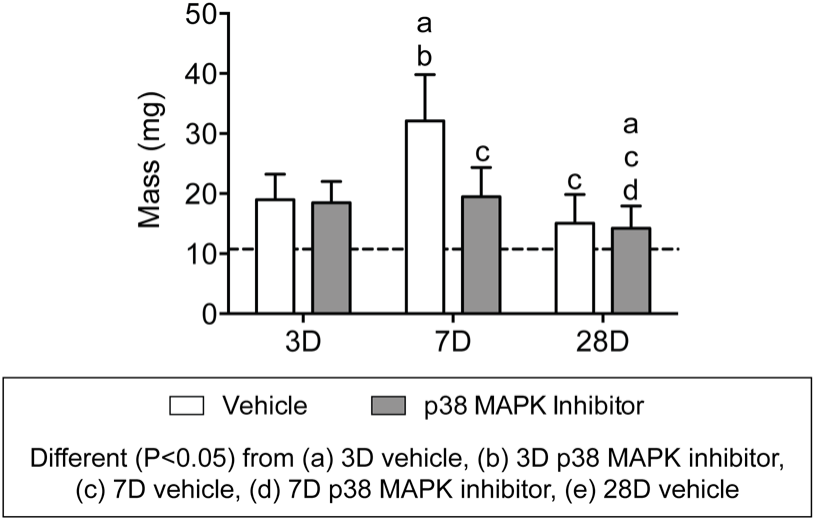
Plantaris tendon mass values. Values are mean±SD, N = 8 for each group. Differences between groups were tested using a two-way ANOVA (α = 0.05). Newman-Keuls post hoc sorting was used to determine differences between groups, and significant interactions (*P*<0.05) are indicated above each group.: a, different (*P*<0.05) from 3D vehicle; b, different (*P*<0.05) from 3D p38 MAPK inhibitor; c, different (*P*<0.05) from 7D vehicle; d, different (*P*<0.05) from 7D p38 MAPK inhibitor; e, different (*P*<0.05) from 28D vehicle. For reference, horizontal dashed line indicates wet mass of plantaris tendons that were not subjected to synergist ablation.

Compared to non-overloaded rats, tenectomy of the Achilles tendon led to a near doubling of tendon mass 3 days after overload (Fig. 2). Between 3 and 7 days after overload, there was a 40% increase in tendon mass in the vehicle group, however no significant differences in tendon mass were observed in this same period for the group that received the p38 MAPK inhibitor. Tendon mass decreased from 7 to 28 days for both vehicle and p38 MAPK inhibitor groups, but remained approximately 30% higher than the mass of non-overloaded plantaris tendons.

**Fig 3:**
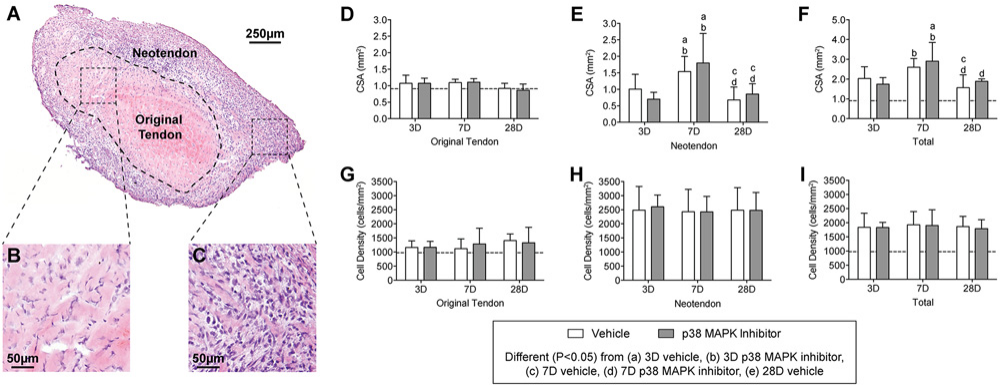
Representative cross-sections of plantaris tendons subjected to synergist ablation, stained with hematoxylin and eosin. (A) Low magnification view of the tendon, and high magnification views of (B) the original tendon and (C) the neotendon are shown. (D-I) Quantitative analysis of tendon sections: Cross-sectional area (CSA, in mm^2^) of (D) the original tendon, (E) the neotendon, and (F) the total tendon. Cell density (cells/mm^2^) of (G) the original tendon, (H) the neotendon, and (I) the total tendon. Values are mean±SD. N, 4 to 8 tendons for each group. Differences between groups were tested using a two-way ANOVA (α = 0.05) followed by Newman-Keuls post hoc sorting: a, different (*P*<0.05) from 3D vehicle; b, different (*P*<0.05) from 3D p38 MAPK inhibitor; c, different (*P*<0.05) from 7D vehicle; d, different (*P*<0.05) from 7D p38 MAPK inhibitor; e, different (*P*<0.05) from 28D vehicle. For reference, horizontal dashed line indicates wet mass of plantaris tendons that were not subjected to synergist ablation.

Morphologically, overload of the plantaris tendon generated a neotendon matrix that arose outward from the most superficial layers of the original tendon (Fig. 3A-C). Other than differences in CSA and density, p38 MAPK inhibition did not impact the general morphological features of tendons, and representative images from a control 3 day overloaded tendon are provided (Fig. 3A-C). For the original tendon region, the CSA of all groups was similar to non-overloaded tendons, and did not change in response to time after overload or treatment with p38 MAPK inhibitor (Fig. 3D). The CSA of the neotendon increased by greater than 50% in both vehicle and treatment groups by 7 days, but decreased by a similar magnitude for both groups between 7 and 28 days (Fig. 3E). Changes in whole tendon CSA generally followed the trends observed in the neotendon data (Fig. 3F). Neither time after overload nor treatment with p38 MAPK inhibitor impacted cell density overall, although the density of cells in the neotendon was considerably greater than what was observed in the original tendon (Fig. 3G-I).

**Fig 4:**
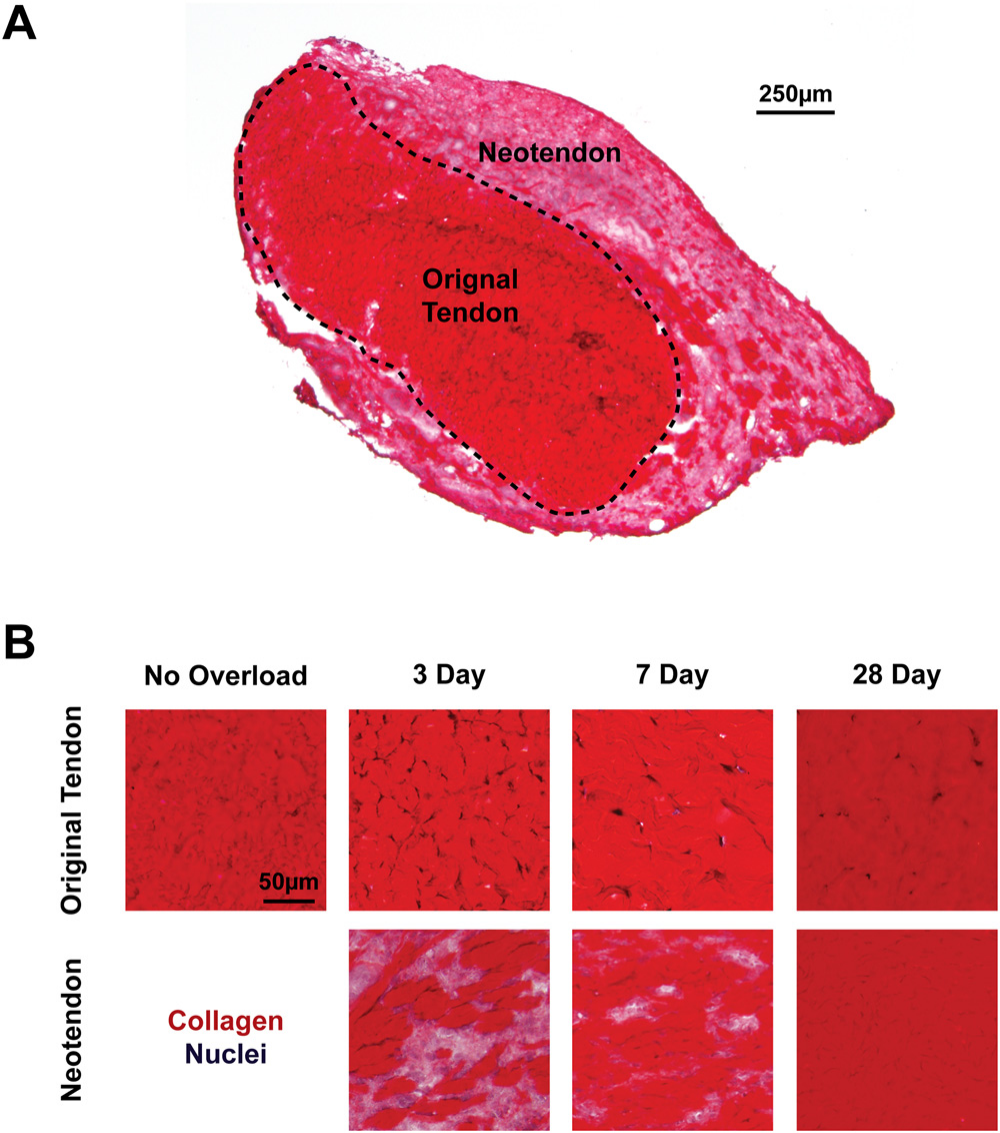
Collagen localization in overloaded tendons. Representative cross-sections of tendon stained with picrosirius red and hematoxylin are provided. (A) Low-magnification image of a 3 day overloaded plantaris tendon demonstrating the differences in collagen density between the original tendon and neotendon. (B) High-magnification images of sections from non-overloaded and overloaded tendons demonstrating the progressive accumulation of collagen in the neotendon following overload. Scale bar is 250μm for (A), and 50 μm for all panels in (B).

**Fig 5:**
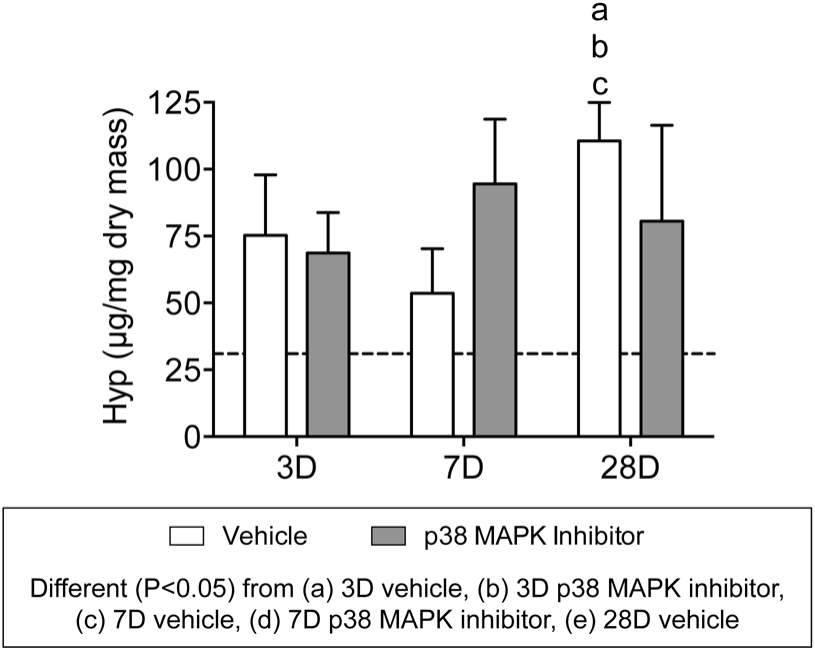
Hydroxyproline content of overloaded tendons. Values are mean±SD. N, 4 to 8 tendons for each group. Differences between groups were tested using a two-way ANOVA (α = 0.05) followed by Newman-Keuls post hoc sorting: a, different (*P*<0.05) from 3D vehicle; b, different (*P*<0.05) from 3D p38 MAPK inhibitor; c, different (*P*<0.05) from 7D vehicle; d, different (*P*<0.05) from 7D p38 MAPK inhibitor; e, different (*P*<0.05) from 28D vehicle. For reference, horizontal dashed line indicates the hydroxyproline content of plantaris tendons that were not subjected to synergist ablation.

After measuring changes in mass and cell density, we next sought to assess changes in collagen content of tendons (Fig. 4A-B). We used picrosirius red staining to identify fibrillar collagens, and similar to what was observed in hematoxylin and eosin stains, p38 MAPK inhibition did not have an effect on gross tendon morphology, so representative images from vehicle treated tendons are shown at each time point. Dense staining of collagen was observed in the original tendon at all time points after overload, and other than differences in cell density, the general features of the collagen was similar to non-overloaded tendons. In the neotendon, however, collagen was present in small pockets 3 days after overload, and gradually increased until nearly the entire neotendon was filled with collagen (Fig. 4B). To perform quantitative measurements of collagen content, we measured levels of the fibrillar collagen marker hydroxyproline. As shown in Fig. 5, the hydroxyproline content of overloaded tendons was approximately 60–200% greater than non-overloaded tendons. With the exception of an increase in hydroxyproline that was observed in the vehicle treated tendons 28 days after overload compared to both 3 day groups and the 7 day control group, there was no effect of time or treatment on hydroxyproline content of tendons (Fig. 5).

**Fig 6:**
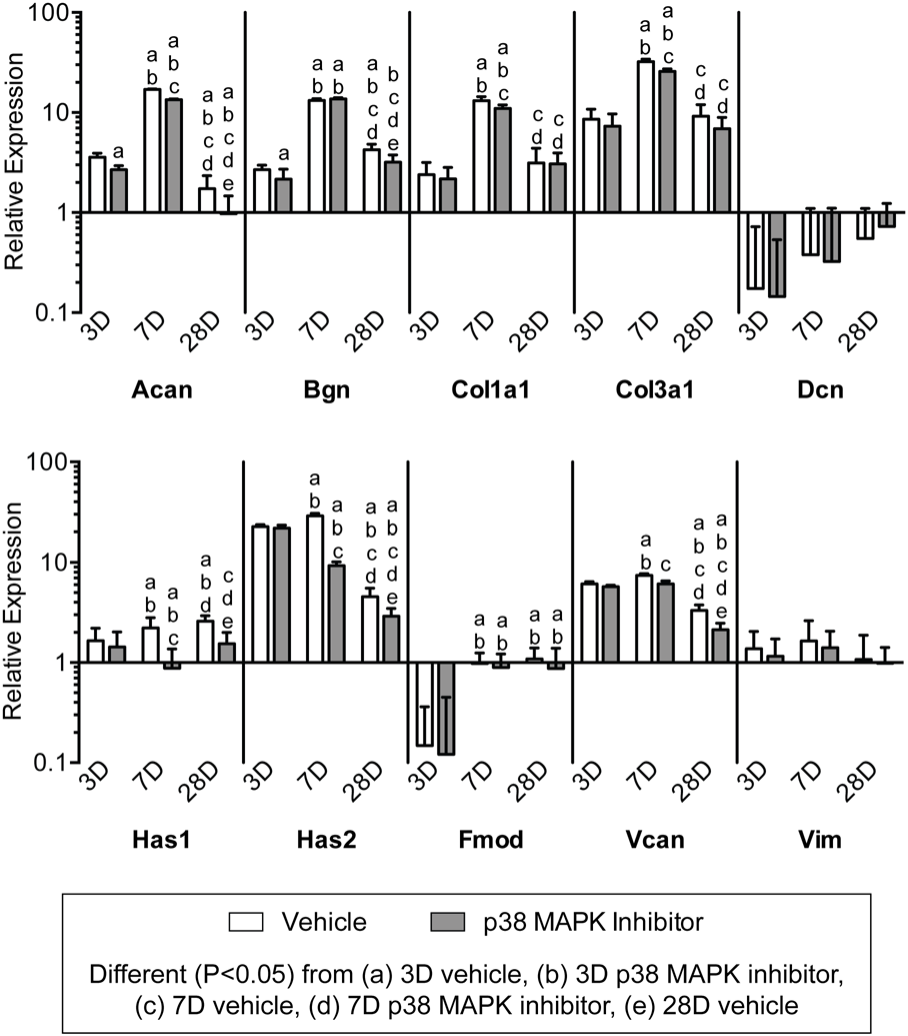
Expression of ECM component genes. Target gene expression was normalized to the stable housekeeping gene beta 2 microglobulin (B2M), and further normalized to plantaris tendons that were not subjected to synergist ablation. Values are mean±SD, N, 8 tendons for each group. Differences between groups were tested using a two-way ANOVA (α = 0.05) followed by Newman-Keuls post hoc sorting: a, different (*P*<0.05) from 3D vehicle; b, different (*P*<0.05) from 3D p38 MAPK inhibitor; c, different (*P*<0.05) from 7D vehicle; d, different (*P*<0.05) from 7D p38 MAPK inhibitor; e, different (*P*<0.05) from 28D vehicle.

**Fig 7:**
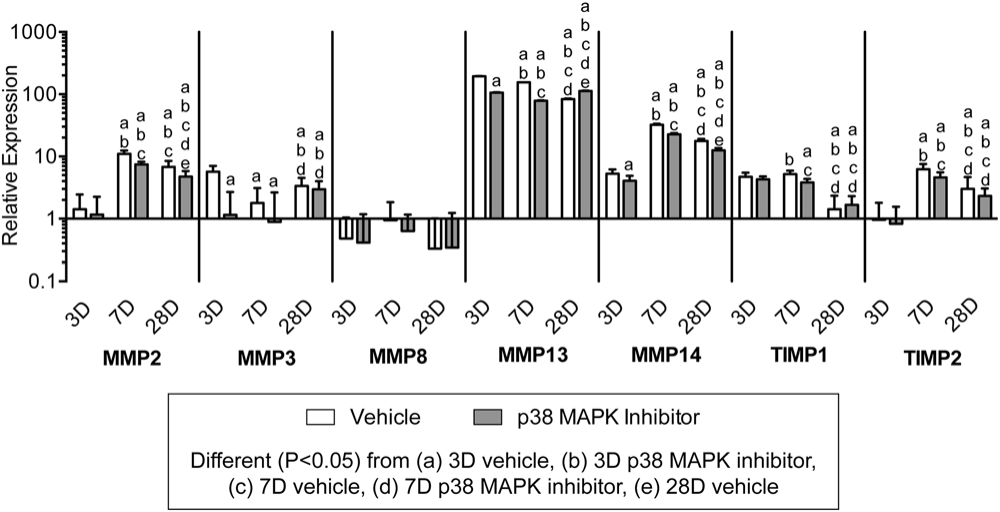
Expression of MMP and TIMP genes. Target gene expression was normalized to the stable housekeeping gene beta 2 microglobulin (B2M), and further normalized to plantaris tendons that were not subjected to synergist ablation. Values are mean±SD, N = 8 tendons for each group. Differences between groups were tested using a two-way ANOVA (α = 0.05) followed by Newman-Keuls post hoc sorting: a, different (*P*<0.05) from 3D vehicle; b, different (*P*<0.05) from 3D p38 MAPK inhibitor; c, different (*P*<0.05) from 7D vehicle; d, different (*P*<0.05) from 7D p38 MAPK inhibitor; e, different (*P*<0.05) from 28D vehicle.

To further evaluate changes in tendon ECM after overload, we measured the expression of genes related to ECM composition and remodeling, and generally observed an effect of both time following overload and p38 MAPK inhibition (Figs. 6 and 7). Transcripts for the HAS enzymes, which synthesize the immature ECM scaffolding hyaluronic acid, were affected by both time after overload and p38 MAPK inhibition. In the vehicle group, the expression of the HAS genes was greatest 7 days after overload, and p38 MAPK inhibition markedly decreased the expression of both transcripts at 7 days and 28 days. The proteoglycan genes aggrecan (Acan), biglycan (Bgn), and versican (Vcan), which are typically enriched at the enthesis, generally followed the same trends observed for the HAS enzymes, although the expression of decorin (Dcn), fibromodulin (Fmod) and the intermediate filament vimentin (Vim) displayed little to no effect of time nor treatment. Type I (Col1a1) and Type III (Col3a1) collagen, which are the major structural proteins of tendon, had the greatest level of expression at 7 days. Inhibition of p38 MAPK did not impact type I or type III collagen expression at 3 or 28 days, although there was a slight decrease in expression for both transcripts at the 7 day time point. Genes controlling members of the matrix metalloproteinase (MMP) and tissue inhibitors of metalloproteinase (TIMPs) families were influenced overall by time following synergist ablation and the inhibition of p38 MAPK (Fig. 7). Transcripts for MMP2 and MMP14 were of highest abundance at 7 days, and p38 MAPK inhibition significantly decreased both transcripts at the 7 and 28 day time points. MMP3 was expressed at its greatest at 3 days in the vehicle group, but its expression remained largely unchanged with p38 MAPK inhibition until increasing significantly at the 28 day time point. There were no differences in MMP8 expression across treatment or time. MMP13 was highly upregulated at all time points compared to non-overloaded tendons, with an overall modest reduction in expression over time, and a negative effect of p38 MAPK inhibition at 3 and 7 days following overload. For TIMP1 and TIMP2 transcripts, expression was maximal at 7 days in the vehicle group but remained significantly downregulated with p38 MAPK inhibition at that time point.

We next sought to identify different types of cell populations present in overloaded tendons. We used immunohistochemistry to determine the presence of these cells (Fig. 9), and gene expression analysis to determine quantitative changes in the markers for these cells and in the factors that regulate their activity (Figs. 9–11). Representative immunohistochemistry from 7 day vehicle treated samples are shown in Fig. 9, although these cells and markers were found in all groups throughout the course of overload. Ki67 is a marker of proliferating cells, CD146 is a marker of pericytes, and F4/80 is a marker of macrophages, and cells expressing all three markers were observed in the neotendon but not in the original tendon (Fig. 9). Type I procollagen is a marker of fibroblasts, and was observed in both the original tendon and neotendon (Fig. 9). Hyaluronic acid, identified with the use of biotinylated hyaluronic acid binding protein (HABP), was found in areas immediately surrounding fibroblasts in the original tendon, but was found throughout the matrix of the neotendon (Fig. 9). For quantitative measures of genes involved in cell proliferation (Fig. 9), Ki67 expression was greatest in both vehicle and treatment groups at 3 days, and steadily decreased over time. CD146 expression was greatest at 7 days, and decreased with p38 MAPK inhibition at the 7 and 28 day time points. Transcripts for the basic helix-loop-helix (bHLH) transcription factor scleraxis (Scx) and the type II transmembrane glycoprotein tenomodulin (Tnmd), which play important roles in fibroblast proliferation and differentiation into the tendon lineage, were both expressed at their greatest at the 7 day time point. Inhibition of p38 MAPK increased scleraxis expression at 7 days and decreased expression at 28 days, but there was no observed influence of treatment on the abundance of tenomodulin transcripts. Hypoxia inducible factor-1 alpha (HIF1a), which can promote fibroblast proliferation, also had generally similar changes in expression with time and treatment. The expression of alpha-smooth muscle actin (SMA), which is a marker of myofibroblasts, was slightly elevated in vehicle and p38 MAPK inhibited groups at 7 days, and returned to baseline levels by 28 days. Transcripts for two other fibroblast markers, fibroblast specific protein-1 (FSP1) and mohawk (Mkx), displayed little to no effect of treatment nor time. Egr1 and Egr2 are transcriptional regulatory proteins involved in tendon development, and while Egr1 was downregulated in all groups compared to non-overloaded samples and displayed no effect of treatment nor time, Egr2 expression peaked at 7 days and was significantly decreased with p38 MAPK inhibition at this time point. The expression of the canonical epithelial-to-mesenchymal transition (EMT) genes Snail1 (Snai1), Slug, Goosecoid (Gsc), and Twist1 were generally greatest at 7 days and remained elevated at all observed time points following overload. The inhibition of p38 MAPK significantly decreased the expression of Snail1 and Twist1 at 3 days, and Snail1, Twist1, and Goosecoid at 7 days.

**Fig 8:**
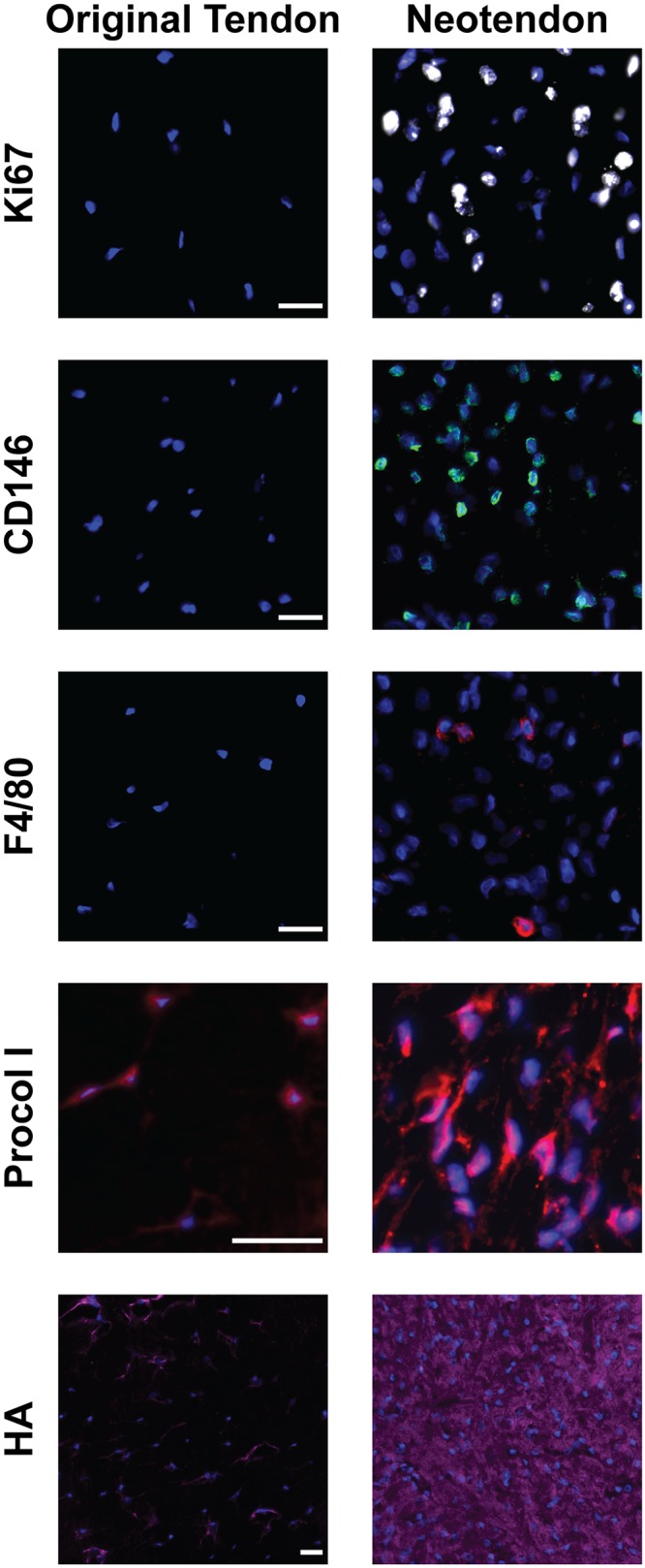
Identification of different cell types and matrix features in overloaded tendons. Representative images of the original tendon and neotendon from 7 day vehicle treated samples demonstrating the presence of proliferating cells (Ki67+, white), pericytes (CD146+, green), macrophages (F4/80+, red) fibroblasts (Procol I+, red), and hyaluronic acid (purple). Nuclei are stained with DAPI (blue). Scale bars are 25μm.

**Fig 9:**
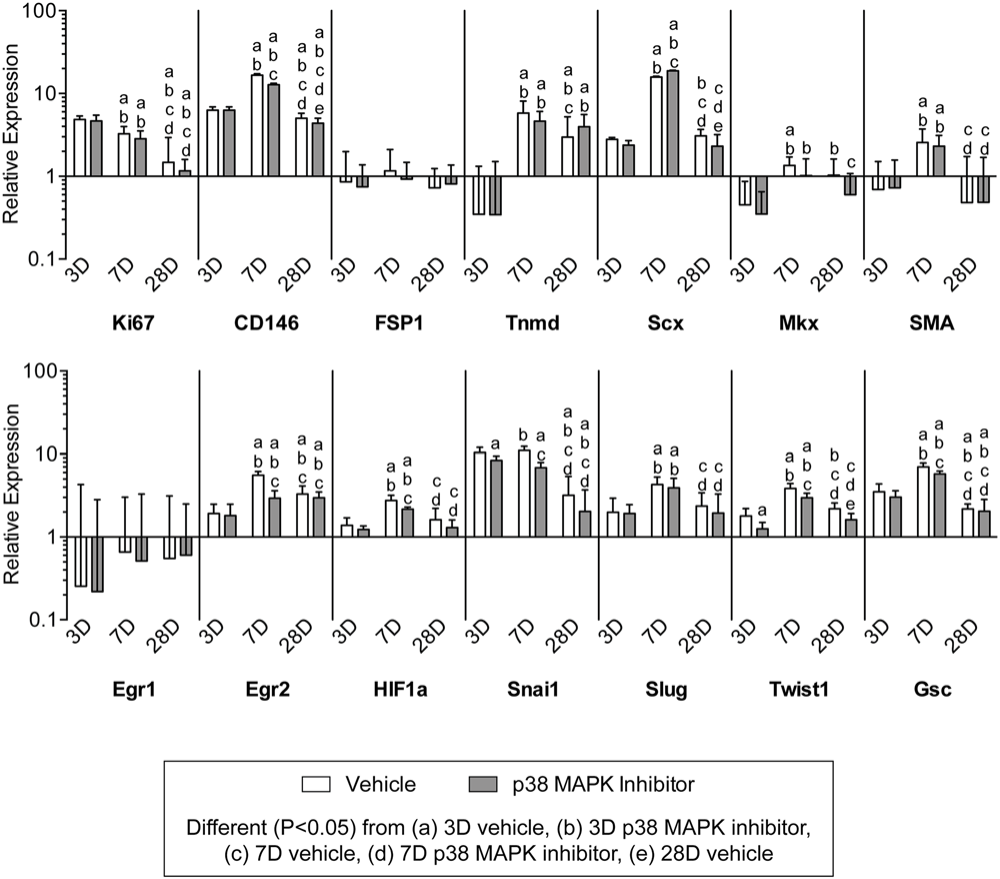
Expression of genes related to cell specification and proliferation. Target gene expression was normalized to the stable housekeeping gene beta 2 microglobulin (B2M), and further normalized to plantaris tendons that were not subjected to synergist ablation. Values are mean±SD, N = 8 tendons for each group. Differences between groups were tested using a two-way ANOVA (α = 0.05) followed by Newman-Keuls post hoc sorting: a, different (*P*<0.05) from 3D vehicle; b, different (*P*<0.05) from 3D p38 MAPK inhibitor; c, different (*P*<0.05) from 7D vehicle; d, different (*P*<0.05) from 7D p38 MAPK inhibitor; e, different (*P*<0.05) from 28D vehicle.

**Fig 10:**
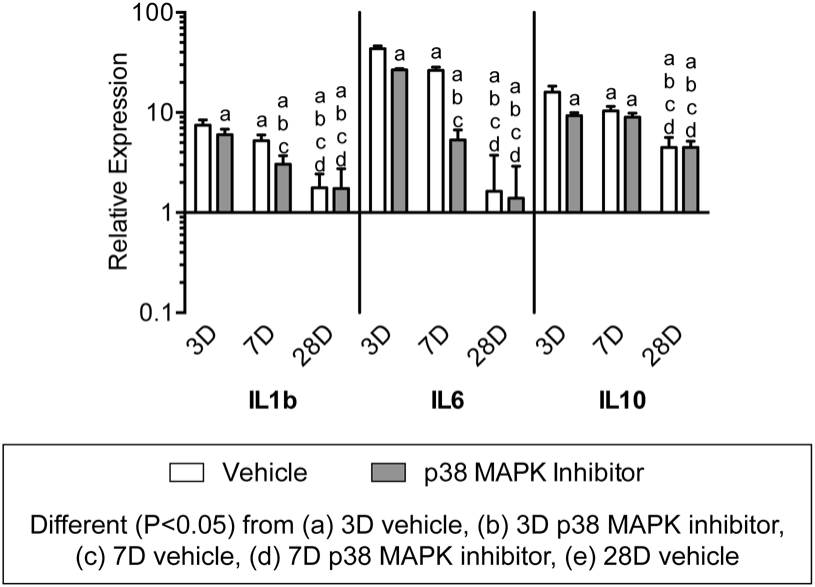
Expression of interleukin genes. Target gene expression was normalized to the stable housekeeping gene beta 2 microglobulin (B2M), and further normalized to plantaris tendons that were not subjected to synergist ablation. Values are mean±SD, N = 8 tendons for each group. Differences between groups were tested using a two-way ANOVA (α = 0.05) followed by Newman-Keuls post hoc sorting: a, different (*P*<0.05) from 3D vehicle; b, different (*P*<0.05) from 3D p38 MAPK inhibitor; c, different (*P*<0.05) from 7D vehicle; d, different (*P*<0.05) from 7D p38 MAPK inhibitor; e, different (*P*<0.05) from 28D vehicle.

**Fig 11:**
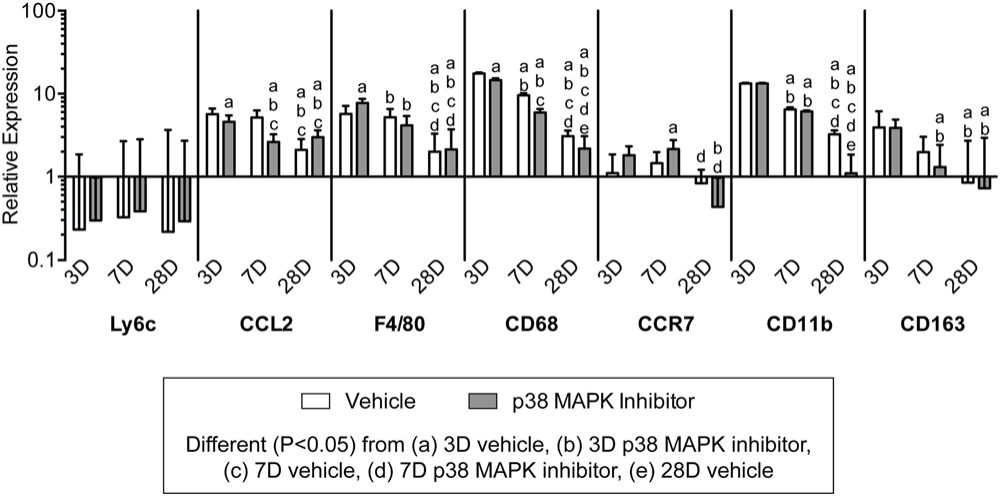
Expression of genes related to neutrophil and macrophage accumulation. Target gene expression was normalized to the stable housekeeping gene beta 2 microglobulin (B2M), and further normalized to plantaris tendons that were not subjected to synergist ablation. Values are mean±SD, N = 8 tendons for each group. Differences between groups were tested using a two-way ANOVA (α = 0.05) followed by Newman-Keuls post hoc sorting: a, different (*P*<0.05) from 3D vehicle; b, different (*P*<0.05) from 3D p38 MAPK inhibitor; c, different (*P*<0.05) from 7D vehicle; d, different (*P*<0.05) from 7D p38 MAPK inhibitor; e, different (*P*<0.05) from 28D vehicle.

We next looked at the expression of genes involved in inflammation and immune cell recruitment. Interleukin genes were dramatically influenced both by time following overload and the inhibition of p38 MAPK. (Fig. 10). The pro-inflammatory interleukins, IL1b and IL6, were maximally expressed in both groups at 3 days and subsequently decreased in expression at the 7 and 28 day time points. The inhibition of p38 MAPK significantly decreased the expression of IL1b and IL6 at 3 and 7 days following synergist ablation. Expression of the anti-inflammatory interleukin, IL10, was elevated at 3 days in both groups but was significantly decreased with p38 MAPK inhibition, and its expression decreased through 28 days in both groups. Ly6c is a marker of neutrophils, and was downregulated compared to control tendons at all time points and appeared insensitive to p38 MAPK inhibition. While no effect was observed for neutrophil markers, inhibition of p38 MAPK and time following synergist ablation influenced the expression of several genes involved in macrophage recruitment and markers of macrophage phenotype (Fig. 11). CCL2, which plays an important role in macrophage recruitment, and the pan-macrophage marker, F4/80, were both expressed at their greatest at 3 days. Inhibition of p38 MAPK significantly reduced the expression of CCL2 and CD68 at 3 and 7 days, and increased the expression of F4/80 at 3 days. The M1 macrophage marker, CCR7 displayed little effect of time nor treatment, and the expression levels were generally similar to non-overloaded tendons. CD11b and CD68 are other markers of M1 macrophages, and both had the greatest level of expression at 3 days, and steadily decreased by 28 days. CD163 is a marker of M2 macrophages, and had the highest expression 3 days after overload and steadily declined thereafter in both vehicle and p38 MAPK inhibitor treated groups.

## Discussion

Hindlimb synergist ablation is a commonly used technique for the study of skeletal muscle hypertrophy in mice and rats [12–18], and we recently demonstrated that this model is also informative to study tendon growth in mice [9]. The main findings from the mouse model of plantaris tendon growth were that fibroblasts in the core of the tendon do not re-enter the cell cycle to contribute to adaptations following overload, and the majority of growth occurs due to expansion of tissue in the outer epitenon/peritenon area of the tendon, which is characterized by an abundance of cells actively undergoing mitosis [9]. The results from the current study support our previous findings in mice, and also provide much greater insight into the mechanisms behind the adaptation of tendon tissue to a mechanical growth stimulus. Further, while the p38 MAPK signaling pathway has previously been shown to play an important role in controlling tendon fibroblast activity in vitro [7, 28], the current study was the first to evaluate the role of p38 MAPK signaling in controlling tendon growth in vivo. The combined results from this study suggest that, as adult tendon grows, the tendon core contributes to neither cellular responses nor structural adaptations, and robust increases in fibroblast proliferation and the infiltration of pericytes enable the tissue to grow from the most superficial layers outward. Furthermore, while inhibiting p38 MAPK modified the expression of several genes as well as tissue mass at 7 days, p38 MAPK signaling does not appear to be necessary for overall tendon growth in vivo.

Some of the most striking changes to tendon following mechanical overload involved components of the tendon ECM. Hyaluronic acid (HA) is an abundant, large glycosaminoglycan that regulates the mechanical properties of ECM and appears to play an active role in promoting cell migration and proliferation [29, 30]. Although scaffolds constructed from HA have been utilized to augment the surgical repair of tendinopathies [31], little is known about endogenous HA and its role in tendon growth in vivo. In skeletal muscle, HA is upregulated during the onset of skeletal muscle hypertrophy, and the HA matrix that is formed directs muscle stem cells to areas of regeneration and assists in the maintenance of these cells in an undifferentiated state [12, 32]. HAS1 and HAS2 are the major enzymes responsible for HA synthesis [33], and in skeletal muscle HAS2 appeared to be the HA synthesis enzyme most sensitive to mechanical loading [12]. In the current study, there was also a marked increase in the expression of HAS2 at the onset of tendon growth, with expression falling substantially by 28 days. In the original tendon, HA was localized immediately adjacent to fibroblasts, while HA was found throughout the neotendon ECM. Despite the rapid and robust increase in HAS2 gene expression by 3 days, the expression of type I collagen and type III collagen, which are the major structural proteins of tendon ECM, did not peak until 7 days after overload. The delay in collagen accumulation in the neotendon could also be observed histologically, with small pockets of collagen apparent 3 days following synergist ablation. By 7 days, the majority of the neotendon matrix stained positive for collagen, and at 28 days nearly the entire neotendon area was occupied by collagen. The dramatic increase in collagen observed histologically is supported by quantitative measures of collagen through hydroxyproline assays. As HA is quite hydrophilic [33], the robust increase in HAS2 expression immediately after overload and subsequent marked decline over time also likely explains the initial increase in tendon wet mass that eventually declines by 28 days. Synergist ablation also appeared to induce robust tissue remodeling. While it is difficult to connect differences in abundance of a specific MMP with changes within the ECM, the stark upregulation in MMP13, and to a lesser extent MMP2, MMP3, TIMP1, and TIMP2 suggest changes in proteolytic activity, that along with increases in collagen expression and hydroxyproline content suggest an active matrix remodeling process. Overall, when taking the phenomena of HA synthesis and changes in collagen metabolism into consideration, the results from the current study suggest that HA is accumulating in the newly forming ECM, and likely serves as a template for cell migration, collagen synthesis, and remodeling.

The mechanical growth stimulus in the current study triggered the accumulation of several cell types in the neotendon. Consistent with results observed in mice [9], actively proliferating cells were restricted to the neotendon, with no proliferating cells present in the original tendon. Macrophages were also present at a low level in the neotendon, and the expression pattern of markers of macrophage phenotype suggested a predominantly pro-inflammatory phenotype at the onset of overload that resolved over time. The majority of the cells observed in both the original tendon and neotendon at all time points stained positive for procollagen type I, suggesting these cells are fibroblasts [34]. However, as the existing fibroblast populations within the original tendon did not appear to re-enter the cell cycle, another population of cells likely served as progenitors for these new fibroblasts in the neotendon. Using a central tendon defect injury model in rats, Tan and colleagues [35] identified a population of CD146+ pericyte cells that accumulated in the outer layers of the injury area and appeared to participate in tendon ECM regeneration. Pericytes are a type of multipotent stem cell population found in the basement membrane of the vasculature and are capable of differentiating into several different mesenchymal cell types, including fibroblasts [36, 37].

In the current study, pockets of CD146+ cells accumulated in the neotendon only, and had a rounded morphology that was distinct from the appearance of fibroblasts. CD146 gene expression was elevated at 3 days, and peaked at 7 days following overload. Scleraxis, which is a marker of the tendon fibroblast lineage [4], and MMP14, which is the membrane-tethered MMP chiefly responsible for fibroblast migration [38], displayed expression patterns similar to CD146. The transcription factors Egr1 and Egr2 play important roles in embryonic tendon development and in tendon regeneration following injury or in response to reloading after immobilization [26, 39–41]. In the current study, to our surprise, Egr1 expression was consistently lower in overloaded tendons than in non-overloaded tendons. This may be due to the acute and transient nature of Egr1 expression [39], and it is possible that changes in Egr1 expression occurred prior to the 3 day time point. Egr2 expression, however, was elevated over baseline at 3 days, and continued to increase until 7 days after overload, in a fashion similar to CD146 and scleraxis. Tenomodulin was downregulated at 3 days, and upregulated at 7 and 28 days, consistent with its described role as a marker of late tendon fibroblast differentiation [42]. The patterns observed in CD146, scleraxis, Egr2 and tenomodulin expression, and the ability of pericytes to differentiate into fibroblasts in other tissues [36, 37], suggest a potential for pericytes to enter the tendon fibroblast lineage and contribute to postnatal tendon growth and matrix production. Another potential source of fibroblast progenitors are epithelial cells, through a process of epithelial-to-mesenchymal transition (EMT) [43]. Tendons are surrounded by an epithelial layer of connective tissue [44], and the EMT-related genes Snail1, Slug, Twist1 and Gsc are coordinately expressed during the regeneration of injured tendons [26]. In the current study, Snail1 expression is highly induced 3 days after overload and remains elevated through 7 days. Slug, Twist1, and Gsc, also demonstrate similar temporal changes in gene expression, with maximum levels observed around 7 days. There has been controversy surrounding the theory of EMT and the ability of epithelial cells to transdifferentiate into fibroblasts [45], although Snail1 and other related proteins can also participate in EMT-independent fibroblast specification and proliferation, such as in the differentiation of pericytes into fibroblasts [46]. The exact identity of the fibroblast progenitor cell populations in adult tendons remain unknown, however the results from this study support a possible role for pericytes as a source of new fibroblasts that can contribute to postnatal tendon growth and remodeling.

Mechanical loading activates several signaling cascades in fibroblasts in various tissue types, including the MAPK pathway [47]. We previously demonstrated an important role for p38 MAPK in regulating tendon fibroblast proliferation and ECM synthesis in vitro [7], but no studies to our knowledge have evaluated the role of p38 MAPK signaling in tendon growth and remodeling in vivo. In skeletal muscle, studies in whole animals have demonstrated p38 MAPK controls critical steps in muscle stem cell division [48], and in the regulation of macrophage activity following tissue injury [49]. For the current study, p38 MAPK inhibition had the most profound effect on IL6 expression during the early phases of tendon growth, which is consistent with findings in skeletal muscle tissue [50]. IL6 is produced in Achilles tendons following prolonged exercise [51], and recombinant IL6 infused into the peritendinous tissue of the Achilles tendon increases collagen synthesis to levels observed with exercise [52]. Consistent with these observations, and results from cultured tendon fibroblasts treated with SB203580 [7], inhibition of p38 MAPK in the current study modestly reduced the expression of type I and type III collagen 7 days following overload. HAS1 and HAS2 expression were also suppressed 7 days after overload by p38 MAPK inhibition. As HA is an extremely hydrophilic compound [33], this reduction in HAS expression likely explains the decrease in wet mass observed in the p38 MAPK inhibitor group compared to vehicle 7 days after overload. IL6 can also direct the accumulation of proinflammatory macrophages during tissue regeneration [53], and we observed a downregulation in CCL2 and CD68 expression 3 and 7 days after overload in the p38 MAPK inhibitor treated groups. Despite a clear suppression of p38 MAPK phosphorylation, and a downregulation in one of the main target genes of p38 MAPK signaling, IL6, the inhibition of p38 MAPK had relatively mild effects on several other genes in this study, and no impact on cell density or CSA measurements. These results suggest that while p38 MAPK plays an important role in controlling the remodeling and regeneration of injured tissue, p38 MAPK in isolation likely plays a minimal role in controlling postnatal tendon growth. The relatively minor role that inflammation appears to have in the findings from the current study further suggest that the synergist ablation technique is a useful model in the study of tendon growth and remodeling.

While we provided important insights into the cellular processes and signaling mechanisms responsible for tendon growth, there are several limitations to this study. Although synergist ablation of the gastrocnemius and soleus muscles through bilateral tenectomy of the Achilles tendon is a quick, well-tolerated, and reproducible model of tendon growth, the mechanical load placed on the plantaris tendon is greater than what is observed in normal locomotion. We did not directly measure the abundance of specific cell types using immunohistochemistry, and relied on gene expression analysis to measure markers of these cells throughout the tendon. SB203580 only inhibits the alpha and beta isoforms of p38 MAPK [54], and it is possible that the gamma and delta isoforms have important regulatory roles in tendon. While we measured the expression of many transcripts, it is possible that observed changes in transcript did not reflect changes at the protein level. Finally, while we analyzed changes in tendon growth 28 days after overload, it is possible further remodeling and adaption occurs beyond this time point.

Unlike other musculoskeletal tissues, very little is known about the fundamental cellular and molecular processes that control tendon growth. While various models have been used to study tendon regeneration after injury [26, 35, 40], these models do not directly address tendon growth. Treadmill training has also been used to study tendon growth [10, 55], but it can take several weeks for ECM to accumulate, and the training regimen is dependent upon the motivation and interest of the animal in performing the exercise. The current study provided important insight into the biological mechanisms of tendon growth in vivo, in particular the accumulation of HA and pericytes in the neotendon, and the dispensability of p38 MAPK signaling in postnatal tendon growth. Postnatal tendon growth appears to occur mostly at the outer layer of tendon, in a similar fashion to the way the rings of a tree grow, which is a notion further supported by Heinemeier and colleagues [56] who observed that the core of tendon tissue in humans appears to stop growing after 17 years of age. The synergist ablation model of plantaris muscle growth has provided important insight into fundamental mechanisms of skeletal muscle hypertrophy and stem cell biology [12–18], and we posit this model can be used to gain a better understanding of the molecular mechanisms of tendon growth and remodeling.

## Supporting Information

S1 Table mRNA Transcripts Evaluated by qPCR. Primers for all genes were purchased from Qiagen, with the exception of MMP3, MMP8, and MMP13,which are from Andarawis-Puri and colleagues (DOI: 10.1002/jor.22059).
(DOCX)
