# Supplementary material for: p38 MAPK Signaling in Postnatal Tendon Growth and Remodeling
Source: PLoS One. 2015 Mar 13;10(3):e0120044. doi: 10.1371/journal.pone.0120044 (PMC4359143; doi:10.1371/journal.pone.0120044)
Supplement: S1 Table — Primers for all genes were purchased from Qiagen, with the exception of MMP3, MMP8, and MMP13,which are from Andarawis-Puri and colleagues (DOI: 10.1002/jor.22059). (DOCX) [file pone.0120044.s001.docx]

**Supporting Information**

***Table SI1. mRNA Transcripts Evaluated by qPCR.*** Primers for all genes were purchased from Qiagen, with the exception of MMP3, MMP8, and MMP13,which are from Andarawis-Puri and colleagues (DOI: 10.1002/jor.22059).

| **Gene** | **RefSeq** | **Name** |
| --- | --- | --- |
| Acan | NM_022190 | Aggrecan |
| Bgn | NM_017087 | Biglycan |
| Ccl2 | NM_031530 | Chemokine (C-C motif) ligand 2 |
| Ccr7 | NM_199489 | Chemokine (C-C motif) receptor 7 |
| Cd11b | NM_012711 | Cd11b molecule (Integrin, alpha M) |
| Cd68 | NM_001031638 | Cd68 molecule |
| Cd146 | [NM_023983](http://www.ncbi.nlm.nih.gov/entrez/query.fcgi?CMD=search&DB=gene&term=NM_023983&doptcmdl=Graphics) | Cd146 molecule (Melanoma cell adhesion molecule) |
| Cd163 | NM_001107887 | CD163 molecule |
| Col1a1 | NM_053304 | Collagen, type I, alpha 1 |
| Col3a1 | NM_032085 | Collagen, type III, alpha 1 |
| Dcn | NM_024129 | Decorin |
| Egr1 | NM_012551 | Early growth response 1 |
| Egr2 | NM_053633 | Early growth response 2 |
| Emr1 (F4/80) | NM_001007557 | EGF-like module containing, mucin-like, hormone receptor-like 1 |
| Fmod | NM_080698 | Fibromodulin |
| FSP1 | NM_012618 | S100 calcium-binding protein A4 |
| Gsc | XM_343101 | Goosecoid homeobox |
| Has1 | [NM_172323](http://www.ncbi.nlm.nih.gov/entrez/query.fcgi?CMD=search&DB=gene&term=NM_172323&doptcmdl=Graphics) | Hyaluronan synthase 1 |
| Has2 | [NM_013153](http://www.ncbi.nlm.nih.gov/entrez/query.fcgi?CMD=search&DB=gene&term=NM_013153&doptcmdl=Graphics) | Hyaluronan synthase 2 |
| Hif1a | NM_024359 | Hypoxia-inducible factor 1, alpha subunit |
| Ki67 | [NM_001271366](http://www.ncbi.nlm.nih.gov/entrez/query.fcgi?CMD=search&DB=gene&term=NM_001271366&doptcmdl=Graphics) | Marker of proliferation Ki-67 |
| Il1b | NM_031512 | Interleukin 1 beta |
| Il6 | NM_012589 | Interleukin 6 |
| Il10 | NM_031512 | Interleukin 10 |
| Ly6c | NM_020103 | Ly6-C antigen |
| Mkx | XM_214497 | Mohawk homeobox |
| Mmp2 | [NM_031054](http://www.ncbi.nlm.nih.gov/entrez/query.fcgi?CMD=search&DB=gene&term=NM_031054&doptcmdl=Graphics) | Matrix metallopeptidase 2 |
| Mmp3 | [NM_133523](http://www.ncbi.nlm.nih.gov/entrez/query.fcgi?CMD=search&DB=gene&term=NM_133523&doptcmdl=Graphics) | Matrix metallopeptidase 3 |
| Mmp8 | [NM_022221](http://www.ncbi.nlm.nih.gov/entrez/query.fcgi?CMD=search&DB=gene&term=NM_022221&doptcmdl=Graphics) | Matrix metallopeptidase 8 |
| Mmp13 | NM_133530 | Matrix metallopeptidase 13 |
| Mmp14 | NM_031056 | Matrix metallopeptidase 14 (membrane-inserted) |
| Scx | NM_001130508 | Scleraxis |
| Slug (Snai2) | NM_013035 | Snail homolog 2 (Drosophila) |
| SMA | NM_031004 | Smooth muscle alpha-actin |
| Snai1 | NM_053805 | Snail homolog 1 (Drosophila) |
| Timp1 | NM_053819 | TIMP metallopeptidase inhibitor 1 |
| Timp2 | NM_021989 | TIMP metallopeptidase inhibitor 2 |
| Tnmd | NM_022290 | Tenomodulin |
| Twist1 | NM_053530 | Twist homolog 1 (Drosophila) |
| Vcan | XM_215451 | Versican |
| Vim | NM_031140 | Vimentin |
